# Laboratory Replication of Ostreid Herpes Virus (OsHV-1) Using Pacific Oyster Tissue Explants

**DOI:** 10.3390/v16081343

**Published:** 2024-08-22

**Authors:** Robert W. A. Potts, Tim Regan, Stuart Ross, Kelly Bateman, Chantelle Hooper, Richard Paley, Ross D. Houston, Tim P. Bean

**Affiliations:** 1 The Roslin Institute, Royal (Dick) School of Veterinary Studies, University of Edinburgh, Edinburgh EH25 9RG, UK; 2 Centre for Environment Fisheries, Aquaculture Science (Cefas) Weymouth Laboratory, Dorset DT4 8UB, UK; 3 Centre for Sustainable Aquaculture Futures, University of Exeter, Exeter EX4 4QD, UK

**Keywords:** oyster, tissue culture, explants

## Abstract

Pacific oysters (*Crassostrea* or *Magallana gigas*) are one of the most economically important aquaculture species globally. Over the past two decades, ostreid herpesvirus (OsHV-1) has become a major pathogen of cultured Pacific oysters, resulting in widespread mortality with a global distribution. Experimental use of OsHV-1 is challenging for many reasons, including both complexity of host–pathogen dynamics and a lack of functioning model systems. The goal of this study was to improve the tools available for working with OsHV-1 in both whole animals and in tissue explants established from oysters maintained in controlled laboratory conditions. Tissue explants were taken from oysters originating from two different sources that have different levels of mortality in experimental OsHV-1 infections and were exposed to OsHV-1. A whole-animal infection experiment was run concurrently as a comparison. Quantitative PCR and electron microscopy were used to confirm that the explants were capable of replicating OsHV-1. Furthermore, the quantitative PCR results suggest that the source of the oysters was significant in determining the outcome of infection in the explants, supporting the validity of the explant model for OsHV-1 infection. This tissue explant approach for studying OsHV-1 allows for the control of confounding factors in the disease outcome that is not possible in whole-animal experiments, providing a new tool for the study of OsHV-1 in Pacific oysters.

## 1. Introduction

Ostreid Herpesvirus 1 (OsHV-1) is an important pathogen of Pacific oyster aquaculture and has been the subject of studies worldwide. However, systems for studying host–pathogen interactions of OsHV-1 and Pacific oysters are limited. Experiments have been conducted in the open ocean where they rely upon natural outbreaks and where there are a wide range of factors that can potentially influence the outcome of infection [1]. For example, abiotic factors can play an important role in infection by stressing the host, which can thus reduce ability to prevent or tolerate infection or create conditions ideal for pathogen proliferation [2]. Additionally, younger oysters are most prone to OsHV-1 infection [3,4], and are also more susceptible to changes in salinity and temperature [5,6]. These changes can be difficult to control in open environments or hatcheries, and are usually a function of normal ecosystem variation. A greater level of control has been achieved by infecting whole oysters with OsHV-1 in managed facilities, such as flow through, recirculating, or static aquaria [7,8,9]. Abiotic factors such as temperature and salinity can be tightly controlled using heaters, chillers, or incubators, and artificial or purified seawater [8]. However, it is not possible to completely control for variability. For example, animals are commonly exposed to the naturally occurring microbiota prior to controlled infection experiments and there are few options to control or effectively manipulate commensal microorganisms in whole animals. The interaction between OsHV-1 and facultative pathogens, such as *Vibrio* spp. bacteria, is complex and can play a major role in the outcome of infection, and indeed even if axenic whole animals could be produced these would not be representative of true infection conditions [10,11]. Furthermore, the microbiome of the oyster plays a key role in the health and disease susceptibility of the host [1] and there remains a vast diversity of marine microorganisms that have not been studied which may have an influence in the aetiology of oyster diseases [1,12]. Detecting and removing microorganisms from experimental animals, and thus from recirculating or static environments, can be challenging. Also, the experimental environment often provides ideal conditions for microbe proliferation, further affecting the desired experimental conditions.

A further issue with using whole animals is inconsistency between individuals. A key example of this is behavioural, e.g., oysters infected using a bath infection method may not open and filter the surrounding water in the time between initiation of the infection experiment and the degradation of the OsHV-1 viability (thought to be within 24 h in experimental systems; see results section), resulting in unsuccessful models of infection. Injecting OsHV-1 directly into the adductor muscle has been used to guarantee the ingress of a known quantity of virus, but this is less reflective of natural infections where exposure will likely occur through gill and/or feeding apparatus. Haemocytes are another potential model for OsHV-1 infection, as they play a key role in host response to infection and have been shown to facilitate OsHV-1 replication in vivo [13]. However, haemocyte cultures from Pacific oysters do not proliferate and are short-lived, making them difficult to work with for experiments lasting over 24 h, which are crucial for studies of complete viral life cycles [14]. Haemocytes from the scallop *Chlamys farreri* have been maintained for long enough to show that OsHV-1 can successfully replicate in haemocyte culture, although this has not been applied to *Crassostrea gigas* [15]. Attempts to replicate OsHV-1 in primary cell types other than haemocytes have so far proved unsuccessful [16].

Creating a model that is representative of the disease dynamics that occur in aquaculture systems whilst removing as many confounding factors as possible would be a major addition to the OsHV-1 research toolkit. The ability to control factors such as temperature, pH, microbiology, and salinity in a laboratory setting would allow for better identification of causative factors in the complexity of disease aetiology. The improvement in primary cell cultures in Pacific oyster offers one potential option [17]. Cell culture systems lend themselves to controlling abiotic conditions through the buffering of pH and temperature, as well as simplified and standardised materials. Utilising enclosed, controlled cell culture environments for experimental infections reduces the technical, logistical, and biosecurity challenges that occur when working with infectious agents in in aquaria or natural environments. The ability to exclude or introduce different abiotic and biotic factors could help to elucidate specific aspects and outcomes of the OsHV-1 infection process. Whole oysters can be reared in axenic conditions, but this requires starting at the larval stage [18]. Removing the microbiota from the oyster without too much stress is a challenge that may be overcome with a cell culture approach. With any model system, it is essential to assess the benefits of simplification against any loss of biological relevance to the field situation. This can partially be achieved by reassessing the difference in outcomes between the whole animals and the cell culture model. If successful, this approach could then be applied to different molluscan aquaculture species and the wide range of diseases that impact them [19].

Here, we demonstrate the effective application of ex vivo *C. gigas* culture systems of several different tissues to study OsHV-1 µvar infection in two different populations of Pacific oyster. The ex vivo model enabled us to quantify the level of viral replication in the different tissue types. Use of both in vivo and ex vivo infection models in two oyster populations with different susceptibility to OsHV-1 provided a comparison of the two methods to validate the use of the tissue explant model for OsHV-1 infection.

## 2. Materials and Methods

### 2.1. Preparation of OsHV-1 Stock

OsHV-1 was acquired from a preserved master stock “MS8” (approximately 3 × 10^5^ viral copies μL^−1^) produced by The Centre for Environment, Fisheries and Aquaculture Science (Cefas) laboratory in Weymouth, derived originally from a natural outbreak of OsHV-1 in Poole Harbour in 2015 (see [20] for viral genome analysis). To amplify viral stocks, oysters approximately 20 mm in height (umbo to bill) were anaesthetised in aerated magnesium chloride seawater solution (50 g/L) for 4 h [21]. Oysters with open valves were split into four groups of 12, transferred to 6-well plates, and the adductor muscle injected with a 40-gauge needle. The first group was injected with 50 µL OsHV-1 MS8 stock immediately after thawing, the second group with a 1 in 10 dilution of OsHV-1 MS8 stock, the third group with a 1 in 100 dilution of MS8 OsHV-1 stock, and the final group was injected with artificial seawater (ASW, 35 ppt, 0.22 µm filtered) as a control. Each well was filled with approximately 10 mL of filtered ASW, to ensure the oyster was completely covered with water. Plates were then incubated at 20 °C in a dark incubator. Water was replaced every 48 h. Oysters were checked daily for mortality by tapping the shell with forceps; any animals that remained open were assumed dead or moribund. Moribund or dead oysters were bagged and kept at 4 °C until the end of the experiment, at which point gill and mantle were dissected out, pooled, and then homogenised using a Stuart SHM10 tissue homogenizer and disposable probes. The homogenate was then diluted in ASW (1:1 volume) and filtered in series though 5 µm, 1 µm, 0.45 µm, and 0.22 µm syringe filters. Aliquots of virus were prepared for all subsequent experiments (labelled as master stock 9; MS9). Aliquots of virus and the homogenate control (prepared in the same way but from uninfected oysters) were also mixed with Glycerol (10% final volume) as a cryoprotectant. 

Control animals were held in separate 6-well plates and dissected in the same manner as dead animals but at the end of the experiment. The homogenate was treated in the same way as infected stock and stored as a homogenate control for subsequent infections.

### 2.2. Animal Husbandry

Juvenile oysters (approximately 20 mm in height, exact age unknown) were acquired directly from the nursery systems of two different commercial oyster hatcheries in the UK. Oysters were maintained in a biosecure oyster holding facility until use. Note: animals from this stock were used in the following experiments and also in the explant dissections described later in this section. Small oysters were kept in 15 L Polypropylene tanks in groups of approximately 100 oysters in ASW. Tanks were aerated and oysters were fed an algal paste diet of shellfish diet 1800 (Reed Mariculture, Campbell, CA, USA) according to manufacturers’ recommendation, and water was replaced every two days. Oysters from different sites were held in identical conditions but in separate tanks.

### 2.3. Whole Animal Experimental Infections and Oyster Site Comparisons

Whole-animal infection experiments were conducted to compare the lethality of OsHV-1 to oysters from different sites, with cryopreserved OsHV-1 MS9. Animals were placed into individual wells of 6-well plates with 10 mL ASW. OsHV-1 MS9 aliquots were thawed on ice and used immediately as follows. A total of 30 animals from each site were subjected to a high dose of inoculum with the addition of 100 µL OsHV MS9 (2 × 10^4^ copies per µL), 30 animals from each site were incubated with a lower dose of 10 µL OsHV-1 MS9, 30 animals from each site were incubated with homogenate control material, and 30 animals from each site were incubated in filtered ASW only. This process was carried out for each of the two oyster sources during the same time period and using an identical viral inoculum. Oysters were checked for mortality daily and water was replaced every 48 h. Dead oysters were stored at −20 °C before being disposed of according to the Roslin Institute aquaculture biosecurity measures plan.

### 2.4. Attenuation of Viral Infectivity in Seawater

To test attenuation of viral infectivity in seawater, filtered ASW containing no virus or 4 × 10^3^ copies of virus μL^−1^ (MS8) was incubated in well plates for 24 h or 48 h at 18 °C in 6-well plates in the dark before the addition of oysters (each of the four treatments was replicated 18 times for a total of *n* = 72 oysters). Mortality was assessed daily and compared to the mortality of oysters exposed to the virus immediately after thawing of the OsHV-1 master stock. This infection experiment was performed separately to those above but using identical husbandry conditions.

### 2.5. Explant Infection 

Explants for infection experiments were prepared from juvenile oysters (approximately 20 mm in height—exact age unknown) sourced from the two different commercial hatcheries and maintained as described above. Whole or partial gill, mantle, and adductor muscle tissues were removed from the oysters with as much tissue as possible dissected in a single piece whilst ensuring no contamination from other tissue types remained. At least 50% of the total tissue was required for each explant and samples that were smaller or potentially contaminated with other tissues were discarded and not used. Explants were prepared as per [17] including disinfection in dilute sodium hypochlorite. Samples were then randomly distributed into infection or control groups for each oyster source, and explants were allowed to recover for 24 h in 1 mL oyster media (50:50 ASW:L15—see [17]) in 24-well plates prior to inoculation with virus. OsHV-1 MS9 (2 × 10^4^ copies per µL) was thawed by a short incubation at 20 °C before 10 µL was immediately added to each infection well.

### 2.6. Tissue Fixation and OsHV-1 Quantification

Two small pieces of tissue (1 to 2 mm^3^) were cut from each tissue type and stored in 2% glutaraldehyde in cacodylate buffer for electron microscopy. Remaining tissue was immersed in RNAlater (Invitrogen, Waltham, MA, USA), snap-frozen, and then stored at −80 °C prior to DNA extraction by the Qiagen (Hildem, Germany) DNeasy blood and tissue kit according to the manufacturer’s protocol. Eluted DNA was also used as a template for qPCR, as below. The absolute number of viral copies per µL was calculated from Ct values using a standard curve generated using a known quantity of plasmid containing a cloned target sequence.

### 2.7. Media OsHV-1 Quantification

An amount of 10 µL of media was taken from directly above the tissue explant daily from each well and snap-frozen. Samples were stored at −20 °C. An amount of 1 µL was used directly as a template for OsHV-1 quantification via qPCR. qPCR was performed using an ABI FAST 7500 real time PCR machine using FAM-based probe detection in 20 µL reaction volumes; 10 µL of TaqMan Fast Advanced Master Mix for qPCR with a 1/20,000 dilution of ROX reference dye, 0.5 µL primer OsHV BF (GTC GCA TCT TTG GAT TTA ACA A), 0.5 µL primer OsHV B4 (ACT GGG ATC CGA ACT GAC AAC), 0.5 µL OsHV-1 probe (FAM- TGC CCC TGT CAT CTT GAG GTA TAG ACA A -TAM) [22], 7.5 µL nuclease-free water, and 1 µL media sample were analysed in 96-well plates. A thermal cycling consisted of 3 min at 95 °C followed by 40 cycles of 95 °C for 3 s and 60 °C for 12 s. Ct values were used to calculate viral copies per µL using a standard curve generated from an OsHV-1 amplicon inserted into a plasmid and aliquoted at a known concentration.

### 2.8. Degradation of OsHV-1 DNA

Four different media were used to test OsHV-1 viral DNA degradation over time in a 24-well plate. An amount of 1 mL of ASW, filtered seawater, reverse osmosis (RO) water, or oyster media was aliquoted into 6 wells each for six replicates per medium. An amount of 1 µL of OsHV-1 MS9 was added to each well and mixed gently by pipetting. An amount of 1 µL was taken from the centre of each well and pooled by medium in a microcentrifuge tube. Samples were snap-frozen and stored at −20 °C before quantification. All samples were thawed and analysed simultaneously. An amount of 1 µL was taken from each pooled sample to use as template for quantitative PCR following the steps outlined above.

### 2.9. Transmission Electron Microscopy

Transmission electron microscopy (TEM) was used to confirm OsHV-1 infection in tissue explants. Samples for TEM were selected based on qPCR quantification data, with samples most likely to be infected being chosen for further assessment, alongside control samples. Quality control and preliminary histology screening were completed using semi-thin sections.

Samples were removed from glutaraldehyde and washed twice in 0.1 M sodium cacodylate buffer for 15 min before post fixation in 1% osmium tetroxide for 1 h. Tissues were rinsed in 0.1 M sodium cacodylate buffer and stored in cacodylate buffer overnight. Samples were dehydrated in a graded acetone series (10, 30, 50, 70, 90, 100, 100%) and embedded within Agar 100 resin (infiltrated with 1:2 then 2:1 mixtures of Agar 100 resin/acetone1, and finally 100% Agar 100 resin). Samples were embedded in BEEM capsules and polymerised for 16 h at 60 °C. Semi-thin sections (1–2 µm) were cut from the resulting blocks and stained on glass slides with 1% Toluidine Blue solution. Slides were examined under a 400× power light microscope and samples with characteristic signs of OsHV-1 infection, marginalised chromatin and empty vacuoles, were selected for ultrathin sectioning. Ultrathin sections (70–90 nm) were cut from selected samples and mounted on uncoated copper grids. Grids were stained with aqueous uranyl acetate and Reynolds lead citrate [23]. Sections were examined with a JEOL JEM 1400 transmission electron microscope, and digital images captured using an AMT XR80 camera and AMTv602 software.

### 2.10. Statistical Analysis

Analysis of survival data was performed using Kaplan–Meier survival analysis in GraphPad version 9.3.1. Statistical analysis of qPCR data was completed using MiniTab version 20.3. Generalised linear models with pairwise analysis by Tukey’s test were applied to media viral load and tissue viral load data. 

## 3. Results

### 3.1. Attenuation of Viral Infectivity in Seawater

**Figure 1:**
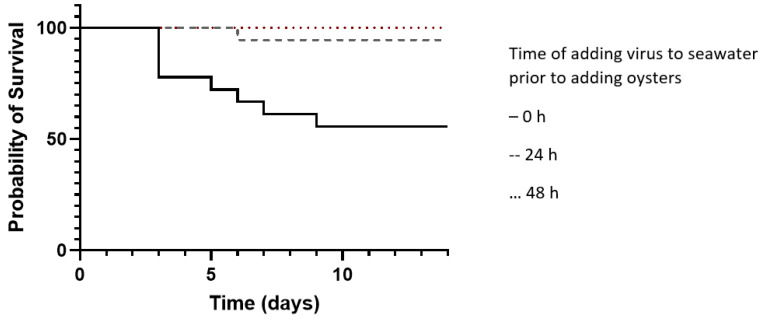
Attenuation of virus-driven mortality in seawater over time. Virus was incubated for 0 (-), 24 (--), or 48 (···) hours prior to addition of oysters (*n* = 18 per treatment) and mortality measured in oysters from this point onwards. Addition at 0 h has significantly different outcome to all other treatments (Log-rank Mantel–Cox test *p* = 0.007).

OsHV-1 showed reduced ability to cause mortality over time spent in seawater, with oyster survival increasing from 66% when virus was added directly alongside oysters, to 94% survival when oysters were added 24 h after virus, and 100% survival following a 48 h interval (Figure 1). No mortalities were observed in seawater controls over the 14-day experiment.

### 3.2. Degredation of Viral DNA in Water 

**Figure 2:**
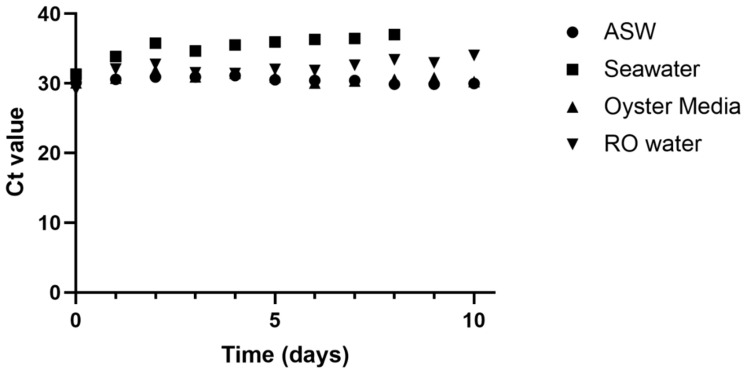
Ct values for viral DNA incubated in ASW, natural seawater, oyster media, and RO water.

OsHV-1 detection by qPCR was consistent at around 30 cycles for ASW and oyster media over the 10-day experiment (Figure 2). Ct values increased slightly over time in RO water, from 29.1 to 34.0, suggesting a decrease in detectable viral DNA. Detection in natural raw seawater showed the greatest decrease, with Ct values rising from 31.3 on day 0 to 37.0 on day 8, after which no OsHV-1 DNA was amplified from the seawater samples. 

### 3.3. Comparison of Mortality from Different Oyster Sources

**Figure 3:**
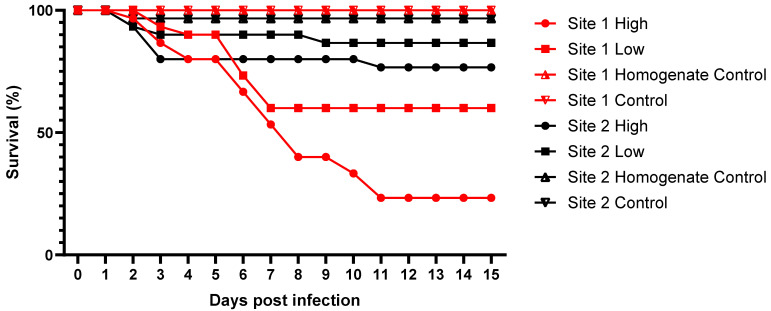
Survival of juvenile oysters from two different sites subjected to a bath infection experiment with a high or low dose of OsHV-1 versus a control (seawater) or a homogenate control (uninfected tissue prepared in the same manner as viral preparation).

Animals from both sites in the seawater control group had 100% survival after the end of the experiment (Figure 3). Survival of animals incubated with oyster homogenate (without OsHV-1) was not significantly different to the control (100% and 97% for site 1 and 2, respectively). Following exposure to the virus, site 1 and site 2 had significantly different survival (Fisher’s exact test *p* < 0.001) across the different test groups, with site 1 having the lowest survival when exposed to a high dose (23%), followed by site 1 low dose (60%), site 2 high dose (77%), and site 2 low dose (87%).

### 3.4. OsHV-1 Replication in Tissue Explant Cultures

**Figure 4:**
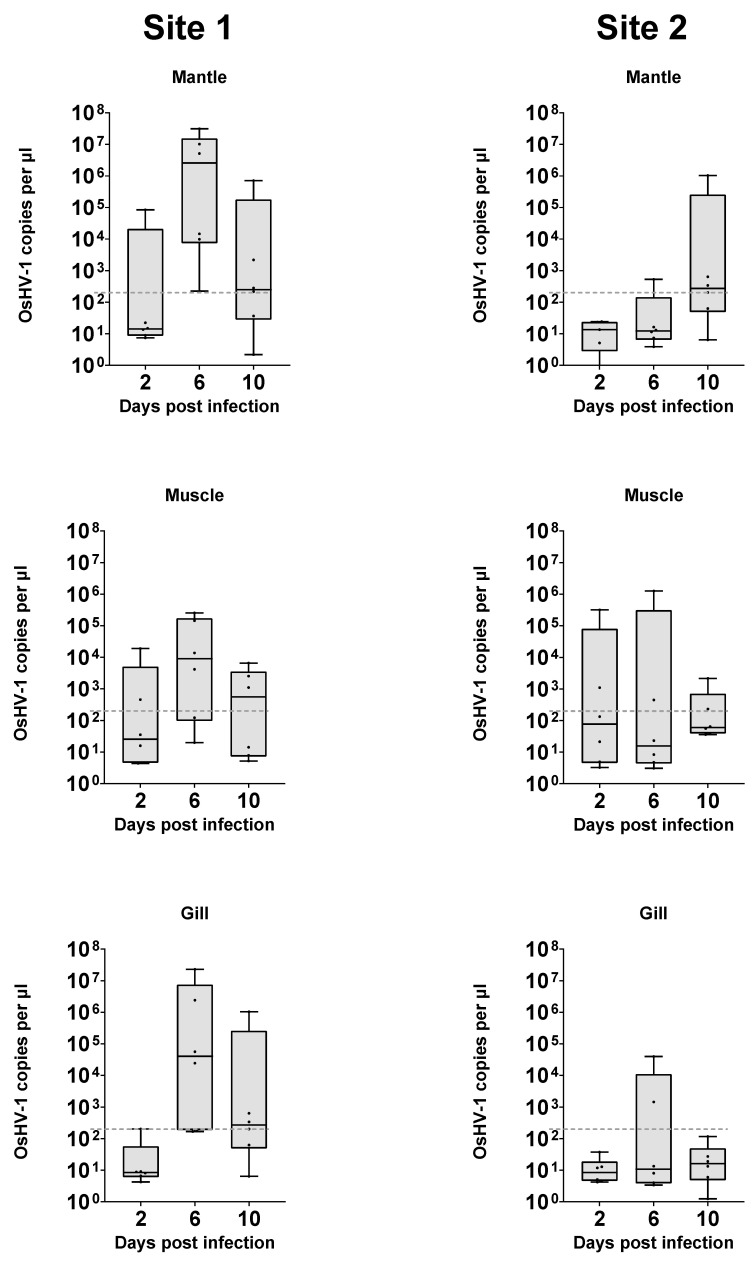
Virus copy number from DNA extraction of mantle, muscle, and gill tissue explants challenged with OsHV-1 over time. Dotted line (----) represents initial viral copy number inoculated into media.

OsHV-1 quantified in DNA extractions from the explanted tissue itself ranged from below the limit of detection to a maximum of 3.1 × 10^7^ copies μL^−1^ of extract. The maximum amount was detected in DNA from a μL of gill sample extract at 6 days post infection and was approximately 100 times greater than the total amount of virus added to each well (2.4 × 10^5^). (Figure 4). As the virus is unable to replicate in media alone (Figure 2), this further demonstrates that OsHV-1 is able to replicate within the tissue explant system developed. When taking all samples into account, a generalised linear model with Tukey’s pairwise comparison demonstrated that viral load was statistically different depending on the source of the oysters (*p* < 0.001), driven mainly by significant differences between the mantle and gill at each time point at each site. Levels of virus in tissues within each site were not significantly different. Over time, there were various notable sample point differences. Day 6 samples from site 1 mantle had significantly more virus than all day 2 and day 10 samples with the exception of site 1 day 10 gill and mantle, which were intermediate in viral load. 

Individual samples were present in each group which had viral loads around or below the limit of detection, suggesting that infection was not consistently present in all samples of any group. These reflect observations in whole-animal infections, where infection is not universal. The frequency at which samples were well above this level was higher in groups from site 1, and at later time points, but these groups are not significantly distinct. 

**Figure 5:**
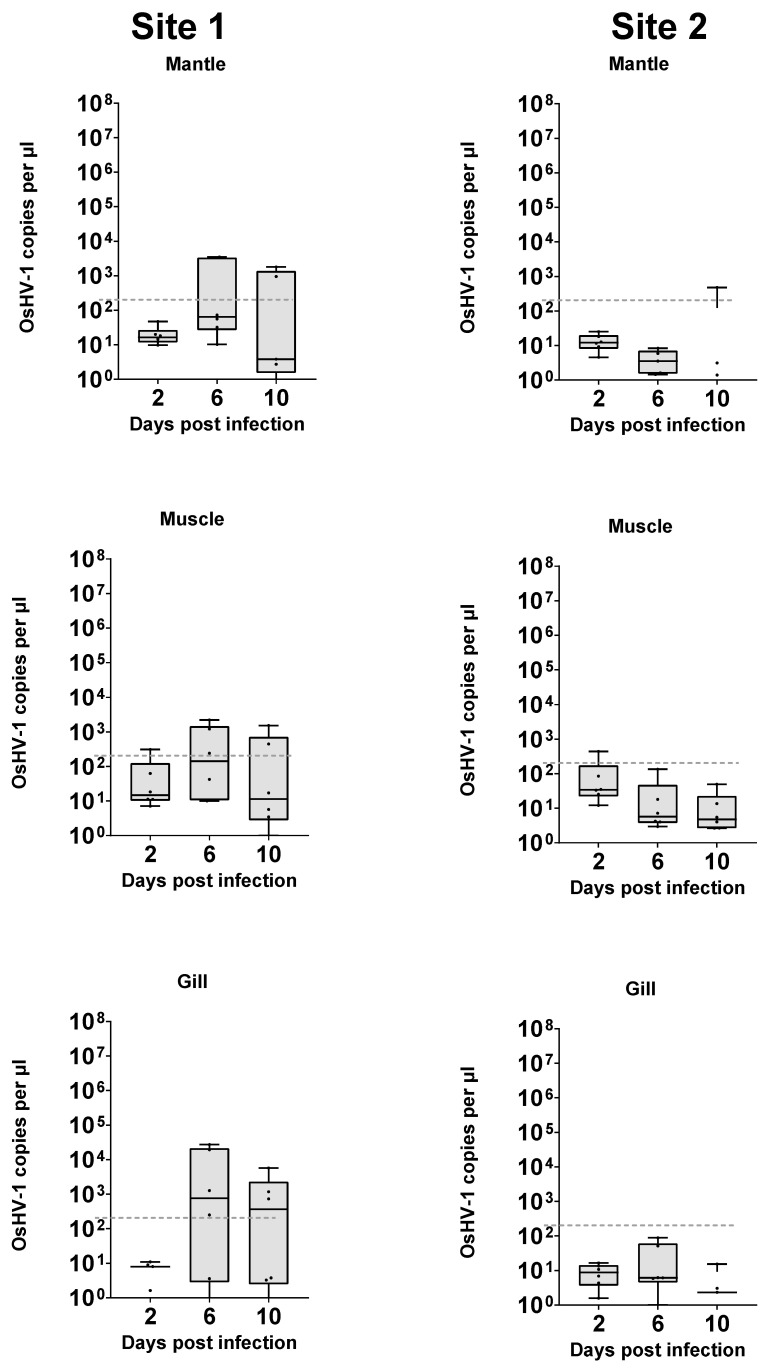
Virus copy number in media surrounding tissue explants challenged with OsHV-1 over time. Dotted line (----) represents initial viral copy number inoculated into media.

OsHV-1 quantification of the media surrounding oyster explants ranged from no detection to a maximum of 2.7 × 10^4^ copies μL^−1^, which was detected in the media surrounding a gill sample from site 1 at 6 days post inoculation (Figure 5). A generalised linear model with Tukey’s pairwise comparisons of viral load of media samples demonstrated a statistically significant difference for source of the oysters (*p* < 0.001), but with no significant difference for days post infection (*p* = 0.076), or for different tissues. The only other statistically distinct group was the day 6 gill group from site 1 (*p* = 0.013), which had a significantly higher level of OsHV-1 DNA detected compared to other groups in the experiment. 

Across all groups, there were samples that had quantifications of less than 10 viral copies per µL, suggesting that infection was not present in all samples from any group. The frequency at which samples were well above this level was higher in groups from site 1, and at later time points. Greater numbers of explants would need to be used to characterise the source of this variability.

### 3.5. Quantification in Tissue VS Media

**Figure 6:**
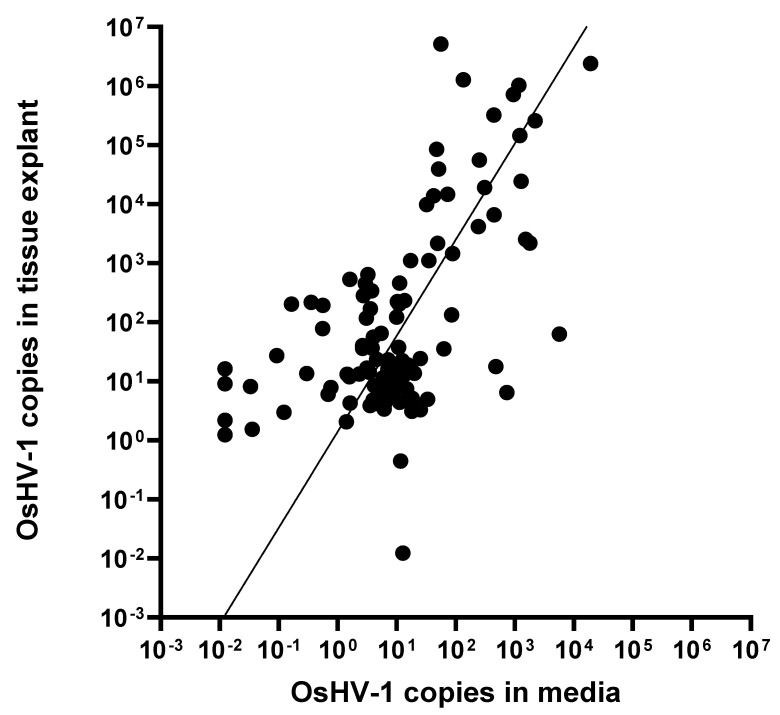
Virus copies in all media compared to values from all tissue DNA extractions. Non-linear regression fit calculated in GraphPad Y = 10^(1.626X + 0.1443)^. Pearsons R^2^ = 0.433, *p* < 0.001.

OsHV-1 was measured in the media itself at days 2, 6, and 10. Copies of viral DNA per ul of media ranged from a high of 2 × 10^4^ to a low of <1, with the majority falling between 10 and 100 (Figure 6). The viral values for detection from DNA extractions taken from tissue samples varied from 10^7^ to <1, with a cluster in the 1 to 1000 range. There is a positive correlation between the ln of viral load values from the media and tissue for each sample (Pearsons R^2^ = 0.433, *p* < 0.001). OsHV-1 detection in the DNA extractions from tissue explants was higher overall than in the media.

### 3.6. Electron Microscopy

**Figure 7:**
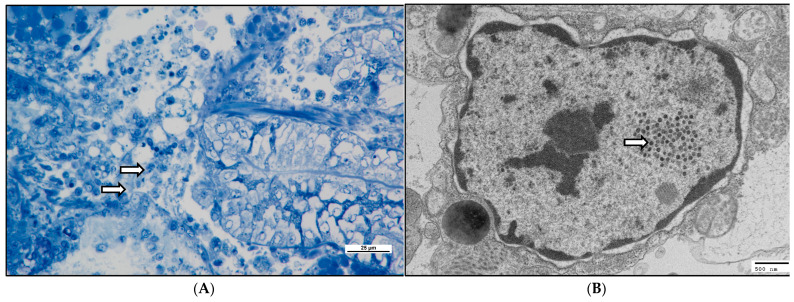
(**A**) Semi-thin section of mantle tissue explant, 6 days post infection. Arrows highlight enlarged nuclei with marginalised chromatin within circulating haemocytes and mantle tissues. Toluidine blue stain, scale bar = 25 µm. (**B**) Electron micrograph of an OsHV-infected sample of mantle tissue 6 days post infection harvested 7 days post explant. Arrow highlights developing virions with the nucleus. Scale bar = 500 nm.

The semi-thin electron microscopy (EM) sections showed marginalised chromatin and empty vacuoles, typical of OsHV-1-infected cells. Marginalised chromatin and empty vacuoles were also visible by TEM, as well as fully formed viral capsids (Figure 7), similar to available OsHV-1 standards (e.g., [16]). 

## 4. Discussion

This research shows that OsHV-1 can replicate ex vivo in tightly controlled laboratory conditions. Samples of explant mantle, muscle, and gill tissues all showed signs of infection in molecular data and we observed pathological evidence of low-level replication in mantle tissues. In addition, no viral replication occurred in the media alone (Figure 2). Replication of OsHV-1 in controlled conditions opens the possibility to study the dynamics of OsHV-1 infection in isolation, without the influence of other biotic factors such as bacteria and animal behaviour, and abiotic factors such as temperature and salinity changes. This approach may be particularly beneficial for studying the role of genetics in disease resistance without confounding factors. Additionally, the tissue explant infection model removes some of the logistical barriers for OsHV-1 research. Oysters can be shipped alive, direct access to seawater is not required, and biosecurity is considerably simpler to attain, so this approach could be carried out anywhere, without requirements for complex aquarium systems. Given further development, ex vivo models may also help to make experiments more consistent and repeatable.

The genotype, life history, and specific physiological conditions of the individual from which the explant was derived are likely to impact the outcome of infection. However, the observed difference in response to infection between the two sources of oysters in whole-animal infection experiment also appears to be reflected in the response of the tissue explants to infection. This suggests that the whole-tissue explant model is relevant to aquaculture environments. The exact factors underlying this difference in susceptibility to OsHV-1 is unknown, but this method will allow for more detailed analysis of underlying mechanisms. Additionally, this finding helps to further validate the usefulness of the large explant method developed previously [17], helping to expand on the range and efficacy of tools available for studying OsHV-1 disease dynamics and marine bivalves generally. However, there was a high level of variability within groups from each location, with some tissues becoming infected and others not. It is unclear yet what this relates to; for example, it could be a natural host response equivalent to that of a resistant animal, a change in proportion of susceptible cells such as haemocytes in an explant, or inefficiencies in the infection model used.

The whole-tissue explant infection model could also potentially help to elucidate the key site of infection within the oyster. Mantle, muscle, and gill tissues were all shown to replicate OsHV-1 in vitro and showed different patterns of viral replication, with the gill and mantle from site 1 (the more sensitive site) showing significantly higher levels of infection than muscle, suggesting they may be more susceptible to initial infection. Haemocytes (free circulating immune cells) have been the focal cell type in OsHV-1 pathology [15,24,25], but as they are found throughout the oyster and in all the tissues examined in this study, it is difficult to confirm that haemocytes are essential in the OsHV-1 lifecycle or if they are infected as a consequence of infection elsewhere in the oyster. It is worth noting that tissue explants will contain a limited number of haemocytes and as such may also have reduced immune response in terms of, for example, lysozyme and antimicrobial peptides compared to whole animals. The impact of this on the outcome will require further clarification. The related Herpes simplex virus (HSV) infects humans and can remain dormant in nerve tissue [26]. Ganglia can be extracted from Pacific oysters, so it may be possible using explants to examine the role of nervous tissue in the OsHV-1 lifecycle [27].

Using OsHV-1 isolated from tissue explants, or by transferring explants into an environment with naïve oysters and monitoring mortality, morbidity, and molecular evidence of infection, would be a useful method to confirm the infectious capabilities of the OsHV-1 produced in explants. This could potentially be used to further examine the dynamics of the infection, including differences between viral replication and shedding in different tissues, from different sources of oyster, or at different times post infection, all studied in isolation with reduced noise compared with conventional in vivo infection models.

An underlying issue with all available models of OsHV-1 infection in oysters is that there is often no way of testing if the oysters used for the infection experiment have previously been exposed to OsHV-1. Unreported OsHV-1 exposure could lead to inadvertently using resistant animals already selected within a population, or potentially the immune priming of animals, making them more resistant to future exposure, thus altering the outcome of infection experiments [28]. It is unclear whether using explants instead of whole animals would help to alleviate this issue. Unreported pathogen exposure may be prevalent across marine molluscs when they are grown in an uncontrolled environment, and the extent to which this plays a role in influencing the results of experimental infection and industrial breeding programs remains unclear. Populations from both sources of oysters studied here seemed to have some level of survival following OsHV-1 exposure, which could be explained by one of these factors, although the current infection status in the UK suggests both source locations are free from OsHV-1. It is currently impossible to tell whether an oyster is completely naïve to OsHV-1 as their immune system is not well characterised for the use of antibody detection, and can be primed by exposure to a different pathogen or physical damage [29,30].

This research also demonstrated the persistence of OsHV-1 DNA in water over time, showing that viral material can be detected in seawater up to 8 days after initial seeding. Interestingly, OsHV-1 DNA degraded less in ASW, RO water, and oyster cell culture media than in natural seawater. The reason for this is unclear, but may be relevant to OsHV-1 monitoring in aquaculture environments. The viability of the virus over time, however, appeared to decay rapidly, and after just 48 h of incubation in artificial seawater it was not able to cause any mortality in oysters. The threshold for virulence has not been thoroughly tested, and is known to vary, so it is possible that there was viable virus present but the amount present was below the critical threshold for this particular experimental system. The reservoir of OsHV-1 during periods without outbreaks has not been identified, but these data suggest it is unlikely to be a free viable virus within salt water systems [31].

## 5. Conclusions

Using the whole-tissue culture system described here, we have shown for the first time that OsHV-1 can replicate ex vivo under controlled laboratory conditions. By using two different sources of Pacific oysters from the UK, quantitative PCR revealed that the phenotype of the donor population is reflected in the outcome of infection for the tissue explants, which supports the model’s relevance to the aquaculture environment, and further validates its use for studying the complexities of Pacific oyster diseases. Improvements in the experimental use and understanding of OsHV-1 have also been described, including the pathogenicity and detectability of virus in experimental systems over time. These advances should help to expand research on OsHV-1 by providing new systems for study.

## Data Availability

Please contact the corresponding author for access to raw data.
